# Editorial: Genetic modulation of gut microbiome: effects on neurological health and aging

**DOI:** 10.3389/fgene.2025.1760164

**Published:** 2025-12-15

**Authors:** Shaohua Wang, Amanda Carroll-Portillo

**Affiliations:** 1 Department of Biomedical Sciences, Ohio University Heritage College of Osteopathic Medicine, Ohio University, Athens, OH, United States; 2 Infectious and Tropical Disease Institute, Ohio University, Athens, OH, United States; 3 Division of Gastroenterology and Hepatology, University of New Mexico, Albuquerque, NM, United States

**Keywords:** aging, genetic tools, gut microbiome, gut-brain, microflora, neurological function

The gut microbiome, an intricate community of trillions of microorganisms, has emerged as a central player in human health, influencing metabolism, immunity, neurological function, and aging (Menezes and Shah, 2024; Sittipo et al., 2022; Rykalo et al., 2024) with rapid advances in genetic technologies such as CRISPR-Cas genome editing and metagenomic sequencing, researchers can now probe and precisely modulate these microbial ecosystems in ways previously unimaginable (Ali et al., 2024; Rahmati et al., 2025). These tools are transforming our understanding of how gut microbes shape the gut–brain axis, neuroinflammation, and age-related cognitive decline, while enabling the development of engineered probiotics and microbiome-based therapeutics. By launching this Research Topic, we aim to bring together diverse perspectives and advances in microbiome science to address how microbial technologies and targeted interventions can be leveraged to prevent and treat human disease. This Research Topic invites contributions that examine microbial mechanisms, microbiome–host interactions, metabolic and neurological conditions, and genetically engineered or modulated gut microbes, as well as strategies that promote healthy aging. Through this call, we seek to engage researchers across microbiology, genetics, metabolism, neuroscience, and translational medicine to define emerging opportunities in microbiome-driven health and therapeutic innovation. To date, the Research Topic features four selected, peer-reviewed contributions, including three original research articles and one review. In the first article, Chen et al. performed a clinical investigation into the gut microbiota of patients with Alzheimer’s disease (AD), applying high-throughput 16S rRNA sequencing to characterize microbial composition and linking these signatures with cognitive performance across different domains. They found that AD patients exhibited significant gut dysbiosis compared to healthy controls, including loss of beneficial, short-chain fatty-acid–producing taxa and expansion of potentially pro-inflammatory bacteria. Importantly, specific microbial features as well as associated metabolite profiles, correlated with impairments in memory, executive function, and other cognitive domains. These results strengthen the hypothesis that gut microbiome alterations may not simply occur alongside AD, but could reflect or contribute to disease severity, offering a basis for microbial biomarkers and microbiome-targeted therapeutic strategies.

The second study, Kunevičius et al. translated this clinical insight into a preclinical model, using the APP/PS1 transgenic mouse model of AD to test whether gut microbiome modulation, via intermittent administration of the beneficial bacterium *Akkermansia muciniphila* together with the prebiotic galactooligosaccharide (GOS), could mitigate disease-related pathologies. Over a prolonged supplementation period, treated mice demonstrated improved metabolic health (e.g., normalized glucose handling), restored gut microbial diversity, balanced short-chain fatty acid (SCFA) levels, and reduced markers of neuroinflammation in the hippocampus. Behaviorally, these mice showed improvements in spatial and recognition memory. Although amyloid-β burden was not significantly reduced, the amelioration of neuroinflammation and improved gut–brain metabolic signaling indicate that microbiome modulation can produce functional benefits independent of direct amyloid clearance. These findings highlight the therapeutic promise of microbiome-based interventions, moving beyond symptomatic management toward modifying disease-associated processes.

In the third contribution, examining a model of Parkinson’s disease (PD), Shan et al. explored how long-term aerobic exercise alters the gut microbiota and whether these changes mediate neuroprotective effects. Using an MPTP-induced PD mouse model, they found that exercise substantially reshaped gut microbial composition and enhanced intestinal barrier integrity. These microbial shifts were associated with upregulation of neurotrophic factors (including BDNF and FNDC5) in the hippocampus, improved synaptic plasticity, preservation of dopaminergic projections, and reversal of cognitive deficits. Crucially, when the gut microbiota was disrupted by antibiotics, the neuroprotective effects of exercise were lost, demonstrating that the beneficial impact of aerobic exercise on neurodegeneration was mediated via the gut–brain axis. This study underscores how lifestyle interventions can harness microbiome plasticity to influence neurodegenerative disease outcomes.

The fourth article, a narrative review from Shi et al., broadens the scope from gut-centric models to a systems-level perspective on neurocognitive disorders associated with aging and physiological stress, notably those following surgery (perioperative neurocognitive disorders, PND). In this narrative review, the authors integrate evidence across multiple organ–brain axes (gut, liver, heart, kidney, immune system, vasculature) to argue that postoperative or age-associated cognitive decline likely results from synergistic dysregulation across these systems. They propose a multi-organs network model: gut dysbiosis, metabolic derangements, vascular insufficiency, immune activation, and organ crosstalk all contribute to neuroinflammation, barrier disruption, and neural vulnerability. This work emphasizes that effective interventions may need to go beyond gut-centric therapies, targeting systemic homeostasis to preserve cognitive health in vulnerable populations.

Collectively, these four studies highlight a converging paradigm (Menezes and Shah, 2024): alterations in the gut microbiome are closely linked to neurological disease and cognitive decline (Sittipo et al., 2022); microbiome-targeted interventions, whether probiotics, prebiotics, exercise, or lifestyle modifications, can reduce neuroinflammation, restore metabolic and gut homeostasis, and improve cognitive performance in preclinical models; and (Rykalo et al., 2024) achieving durable benefits in aging or disease contexts likely requires integrated, multi-system approaches rather than focusing solely on the gut. Together, this body of work underscores the promise of the microbiota–gut–brain axis as a versatile therapeutic target for neurodegeneration, healthy aging, and cognitive resilience, while also emphasizing the importance of systemic, organ-level integration for long-term impact.
